# The association between environmental factors and the development of Crohn’s disease with focusing on passive smoking: A multicenter case-control study in Japan

**DOI:** 10.1371/journal.pone.0216429

**Published:** 2019-06-07

**Authors:** Kyoko Kondo, Satoko Ohfuji, Kenji Watanabe, Hirokazu Yamagami, Wakaba Fukushima, Kazuya Ito, Yasuo Suzuki, Yoshio Hirota, for the Japanese Case-Control Study Group for Crohn's disease

**Affiliations:** 1 Osaka City University Hospital Administration Division, Osaka, Japan; 2 Department of Public Health, Osaka City University Graduate School of Medicine, Osaka, Japan; 3 Department of Intestinal Inflammation Research, Hyogo College of Medicine, Hyogo, Japan; 4 Department of Gastroenterology, Osaka City University Graduate School of Medicine, Osaka, Japan; 5 Inflammatory Bowel Disease Center, Toho University Sakura Medical Center, Chiba, Japan; 6 College of Healthcare Management, Fukuoka, Japan; University of Oxford, UNITED KINGDOM

## Abstract

**Background:**

The number of patients with Crohn’s disease (CD) in Japan has recently been increasing. We examined the association between environmental factors and the development of CD in Japanese focusing on passive smoking.

**Methods:**

We conducted a multicenter case-control study and compared the environmental factors of 93 cases who were newly diagnosed with CD to the environmental factors of 132 controls (hospital-, age-, and sex-matched patients with other diseases). The odds ratio (OR) of each factor for the development of CD and the 95% confidence interval (CI) were calculated using a logistic regression model. The association between the details of passive smoking history and the development of CD was examined for those who had an active smoking history "no". Odds ratios of number of passively smoked cigarettes (per day), time of passive smoking (per day) and period of passive smoking (year) were calculated using “passive smoking ‘No’” as a reference.

**Results:**

History of appendicitis, family history of inflammatory bowel disease, and active smoking history were not significantly associated with the development of CD. Drinking history showed a decreased OR for the development of CD (0.39, 0.19–0.77). "Passive smoking Yes" showed significantly increased OR (2.49, 1.09–5.73). Regarding the association between passive smoking and the development of CD, the OR increased as the number of cigarettes per day, smoking time per day, and smoking duration increased, and there was a dose-response relationship (trend P = 0.024, 0.032, 0.038).

**Conclusions:**

The association between environmental factors and the development of CD among Japanese was examined by case—control study. It was suggested that the passive smoking history may be associated to the development of CD.

## Introduction

The prevalence of Crohn’s disease (CD) in Japan has been increasing over the past 20 years [1], and in 2005, the age-standardized prevalence of CD was 21.2 per 100,000 [2]. The cause of this has not yet been clarified, and while various epidemiological studies have been conducted, few studies have involved Japanese persons. Previous studies have suggested that environmental factors play an important role in the development of CD. There is a report that a history of appendicitis is a risk factor for CD [3, 4], but there is a report stating that it is difficult to clarify [5], and definite conclusions have not been reached. The presence of a family history of inflammatory bowel disease (IBD) in CD patients was significantly lower in China than in the United States [6]. The association between smoking and CD is reported as follows: passive smoking in childhood is associated with the development of CD [7], smoking currently is associated with an elevated risk of CD [8–10], and smoking in patients with CD has an adverse effect [11]. On the other hand, the role of alcohol on inflammatory bowel disease has not been clear[12]. Epidemiologic studies investigating the association between CD and alcohol are very few, but two cohort studies in Europe showed that there is no evidence that there is an association between alcohol intake and UC or CD onset [10, 13].

A case-control study was conducted to clarify factors associated with the development of CD in a Japanese population. Since the number of patients with CD is limited, case-control studies are considered to be suitable for exploring risk factors. However, regarding the patient information at the time of the investigation, there is a possibility that some patients have changed their lifestyle habits following the onset of symptoms due to the disease. Therefore, to maintain the temporal relationship between exposure and outcome, it is necessary to include newly diagnosed CD patients as cases. Furthermore, in order to evaluate the association with the lifestyle before disease as precisely as possible, we collected pre-illness information as much as possible.

## Methods

### 1. Study design and subjects

Between October 2011 and March 2016, a multicenter case-control study was conducted at 45 collaborating hospitals in Japan. Eligible cases were patients who were newly diagnosed with CD at those hospitals and were less than 80 years old at the time of diagnosis. In the case of patients who were referred after confirmation at other hospitals, they could be included if the confirmed diagnosis was within 6 months before the referral visit. The diagnostic criteria of CD were as follows, 1) longitudinal ulcer or paving stone image, or 2) non-toxic epithelial cell granulomas and ambiguous, approximately circular ulcer or extensive afterpattern in the gastrointestinal tract or characteristic anal lesion, or 3) ambiguous, approximately circular ulcer or extensive afterpattern in the gastrointestinal tract, characteristic anal lesion, and distinctive gastric and duodenal lesions [14]. These patients were asked to participate in the study as soon as possible after diagnosis.

Two matched controls for each CD case were selected in the same hospital as the enrolled CD case. One of them was selected from the department of gastroenterology and the other was selected from a different department (Orthopedic Surgery, Ophthalmology, General Medical Department, etc.). Conditions of matching were sex and age (5-year age groups: under 10, 10–14, 15–19, 20–24, …, 75–79). The exclusion criteria were follows: presence of malignant neoplasms; lasting symptoms of diarrhea and/or abdominal pain for more than one week at the time of entry; or history of IBD. Each cooperating hospital was asked to provide 2 sets of these cases and controls each year.

The research protocol was executed according to the Helsinki Declaration with the approval of the Ethics Committee of Osaka City University Graduate School of Medicine. In addition, as necessary, each participating facility was also approved by the Ethics Review Committee. Written, informed consent was obtained from all subjects prior to participation. If the subject was under the age of 20 years, written, informed consent was obtained from the legal representative of the subject.

### 2. Information collection

Using the standardized questionnaire, the following clinical findings of CD patients were reported by the gastroenterologist in charge: date at symptom onsets; date at first visit to the hospital; location of disease at diagnosis (only small intestine, small and large intestine, or large intestine only); and systemic complications. Subsequently, age at symptom onset, the period from symptom onset to study participation, and the period from the first visit to study participation were calculated from the date of birth, date at symptom onset, date at first visit to the hospital, and study participation date. In addition, the subjects were asked to complete a self-administered questionnaire. Contents of the questionnaire were demographic factors, height and weight at examination, past medical history including appendicitis, family history of IBD, drinking history, and smoking history (active smoking history, passive smoking history when no active smoking history). BMI was calculated from each patient’s weight (kg) divided by the square of the height (m^2^).

### 3. Statistical analysis

Each variable was defined as follows: appendicitis history, “yes” was a person who had had appendicitis more than one year earlier; family history of IBD, “yes” was a person with an up to second-degree relative with either ulcerative colitis or CD; drinking history, “yes” was “those who are drinking now” or “drinking in the past (abstinent person)”; active smoking history, “yes” was “those who are smoking now” or “smoking in the past (smoking cessation)”; and passive smoking, “yes” was a person who had exposure to passive smoke in more than one year ago.

The Chi-squared test or Fisher’s exact test was used to compare the characteristics of cases and controls. For the analysis method, a multiple logistic regression model was used to calculate the odds ratio (OR) and 95% confidence interval (CI) of each factor for the development of CD. The variables included in the multivariate model were (1) statistically significant factors in the sex and age-adjustment analysis and (2) medical and biologically meaningful factors regardless of statistical significance.

Next, we examined the relationship between the passive smoking history and the development of CD, for those without active smoking history. Odds ratios of number of passively smoked cigarettes (per day), time of passive smoking (per day) and period of passive smoking (year) were calculated using “passive smoking ‘No’” as a reference. The level of significance was set at P <0.05. For the analysis, SAS Version 9.4 (SAS Institute, Inc., Cary, NC, USA) was used.

## Results

Of the 279 participants (CD case 116, control 163), 241 (case 101, control 140) returned questionnaires (86% response rate), of which 16 had missing values. Therefore, the participants brought some variation in matched status. Forty sets (40 cases, 80 controls) maintained the initial matching condition, but 26 cases had only one corresponding control and 27 cases and 26 controls had no corresponding counterparts for pairing. So, in order to increase the statistical power, main analyses were performed in all 225 participants (case 93, control 132) who responded to the questionnaire, using unconditional logistic regression model with adjustment for matching factors (age categories and gender).

Table 1 shows the clinical characteristics of the newly diagnosed CD cases. The mean age at examination was 30.5 years. Approximately 95% of cases had symptom onset within 6 months. Of cases 21% had disease in the small intestine only, 63% had disease in the small intestine and large intestine, and 16% had disease in the large intestine only.

**Table 1:** Clinical characteristics of newly diagnosed Crohn’s disease cases (N = 93)*.

Characteristic		n	(%)
Sex	Male	68	(73)
	Female	25	(27)
Age at examination (y)	Mean (SD)	30.5	(12.2)
	<20	19	(20)
	20–29	33	(36)
	30–39	19	(20)
	≥40	22	(24)
Age at symptom onset (y)	Mean (SD)	29.9	(11.5)
	<20	14	(25)
	20–29	16	(29)
	30–39	12	(22)
	≥40	13	(24)
	Unknown	38	
Duration from symptom onset (months)	Median (range)	1.2	(0–14.4)
	<7	88	(95)
	7–11	3	(3)
	≥12	2	(2)
Duration from first visit to the hospital (months)	Median (range)	4.8	(0–52.8)
	<7	37	(67)
	7–11	9	(16)
	≥12	9	(16)
	Unknown	38	
Disease sites at diagnosis	Only small intestine	15	(21)
	Small and large intestine	45	(63)
	Large intestine only	11	(16)
	Unknown	22	
Intestinal tract complications	No	49	(69)
	Yes	22	(31)
	Unknown	22	
Extra-intestinal tract complications	No	54	(78)
	Yes	15	(22)
	Unknown	24	

SD, standard deviation

* Data expressed as n (%) unless otherwise indicated.

Controls were selected from the department of gastroenterology and different department, and the ratio was approximately 1: 1. The most frequent digestive disease was liver disease (n = 25), followed by upper digestive disease (n = 17) and colon disease (n = 19). The most frequent diseases from other departments was orthopedic disease (n = 13), followed by cardiovascular disease (n = 8), chronic kidney disease (n = 7) and others (n = 42).

Table 2 shows the background characteristics of the cases and controls. The distributions of sex, age, family history of IBD, and active smoking history were similar between cases and controls. However, the cases had a lower BMI at examination and a more frequent history of appendicitis. Furthermore, a significant difference was observed in drinking history between the two groups.

**Table 2:** Characteristics of the cases and controls.

			Cases		Controls		P*
			(N = 93)		(N = 132)		
			n	(%)	n	(%)	
Sex							
	Male		68	(73)	86	(65)	0.205
	Female		25	(27)	46	(35)	
Age (y)							
	<20		19	(20)	31	(24)	0.734
	20–29		33	(35)	41	(31)	
	30–39		19	(20)	33	(25)	
	≥40		22	(24)	27	(20)	
BMI at examination (kg/m^2^)							
	<18.5		27	(29)	19	(14)	0.0004
	18.5–24.9		59	(63)	84	(64)	
	≥25.0		7	(8)	29	(22)	
History of appendicitis							
	No		81	(87)	126	(95)	0.023
	Yes		12	(13)	6	(5)	
Family history of IBD							
	No		88	(95)	128	(97)	0.494
	Yes		5	(5)	4	(3)	
Drinking history							
	No		54	(58)	59	(45)	0.048
	Ever or Current		39	(42)	73	(55)	
Smoking history							
	Active smoking No						
		Passive smoking No	35	(38)	66	(50)	0.163
		Passive smoking Yes	20	(22)	20	(15)	
	Active smoking (ever or current) Yes		38	(41)	46	(35)	

BMI, body mass index; IBD, inflammatory bowel disease

*The χ^2^ test or Fisher’s exact test was used as appropriate.

Table 3 shows the association between environmental factors and the development of CD. Sex, age, BMI, history of appendicitis, IBD family history, drinking history, smoking history were included in the multivariate model. A history of appendicitis showed a significant increase in the sex, age-adjusted OR (3.12, 1.09–8.92), but the adjusted OR (2.26, 0.74–6.89) was not significant. A family history of IBD was not significantly associated with the development of CD (1.86, 0.41–8.35). Those who had history of drinking had a significant decrease in OR for the development of CD (0.39, 0.19–0.77). Regarding smoking history, when reference category was "no active smoking, no passive smoking", the OR of "no active smoking, with passive smoking" was significantly increased (2.49, 1.09–5.73), but the OR of "with active smoking" did not reach significance (1.82, 0.85–3.92).

**Table 3:** Odds ratios of environmental factors for development of Crohn's disease.

	No. cases/controls			Sex, age-adjusted			Multivariate*		
				OR	(95%CI)	P	OR	(95%CI)	P
History of appendicitis									
	No		81/126	1.00			1.00		
	Yes		12/6	3.12	(1.09–8.92)	0.034	2.26	(0.74–6.89)	0.152
Family history of IBD									
	No		88/128	1.00			1.00		
	Yes		5/4	2.03	(0.51–8.00)	0.313	1.86	(0.41–8.35)	0.419
Drinking history									
	No		54/59	1.00			1.00		
	Ever or Current		39/73	0.39	(0.21–0.74)	0.004	0.39	(0.19–0.77)	0.007
Smoking history									
	Active smoking No								
		Passive smoking No	35/66	1.00			1.00		
		Passive smoking Yes	20/20	1.93	(0.91–4.11)	0.086	2.49	(1.09–5.73)	0.031
	Active smoking (ever or current) Yes		38/46	1.51	(0.78–2.94)	0.223	1.82	(0.85–3.92)	0.124

OR, odds ratio; CI, confidence interval; IBD, inflammatory bowel disease

* Model included sex, age, BMI, history of appendicitis, family history of IBD, drinking history, and smoking history.

Next, we show the association between passive smoking and the development of CD (Table 4). The risk increased as the number of cigarettes smoked by nearby smokers increased (trend P = 0.024), and became significant at 15 or more cigarettes (3.15, 1.10–9.06). The risk increased with longer passive smoking time per day (trend P = 0.032) and became significant at over more than 4 hours (2.77, 1.02–7.56). The risk increased as the period of passive smoking increased (trend P = 0.038), indicating marginal significance at over 17 years (2.68, 0.91–7.89).

**Table 4:** Association between passive smoking and the development of Crohn's disease.

		Cases (N = 55)		Controls (N = 86)		Sex, age-adjusted			Multivariate model*		
		n	(%)	n	(%)	OR	(95%CI)	P	OR	(95%CI)	P
Number of passively smoked cigarettes (per day)											
	No	35	(64)	66	(77)	1.00			1.00		
	<15	8	(15)	10	(12)	1.53	0.55–4.27	0.418	1.95	(0.66–5.79)	0.230
	≥15	12	(22)	10	(12)	2.29	0.89–5.89	0.087	3.15	(1.10–9.06)	0.033
						(Trend P = 0.074)			(trend P = 0.024)		
Time of passive smoking (hours per day)											
	No	35	(64)	66	(77)	1.00			1.00		
	<4	8	(15)	9	(10)	1.65	0.58–4.73	0.352	2.17	(0.69–6.82)	0.185
	≥4	12	(22)	11	(13)	2.12	0.84–5.38	0.113	2.77	(1.02–7.56)	0.046
						(Trend P = 0.088)			(trend P = 0.032)		
Period of passive smoking (y)											
	No	35	(64)	66	(77)	1.00			1.00		
	<17	10	(18)	10	(12)	1.91	0.72–5.09	0.195	2.35	(0.81–6.78)	0.115
	≥17	10	(18)	10	(12)	1.90	0.71–5.08	0.200	2.68	(0.91–7.89)	0.073
						(Trend P = 0.119)			(trend P = 0.038)		

OR, odds ratio; CI, confidence interval

* Adjusted by sex, age, BMI, history of appendicitis, family history of IBD, and drinking history.

## Discussion

This case-control study showed an association between passive smoking and the development of CD. In the passive smoking, the OR increased as the number of cigarettes per day, smoking time per day, and smoking duration increased, and there was a dose-response relationship. However, although the OR of active smoking for the development of CD increased, it was not significant.

Active smoking has also been reported as a risk factor for CD in previous studies. In case-control studies in Italy [8] and Sweden [10], active smokers had significantly increased ORs for CD compared with nonsmokers. In a prospective study of American women, the hazard ratio of smokers was 1.90 (1.42–2.53) [9]. With respect to passive smoking, there is a report that passive smoking in childhood affects the progression of CD [7, 15]. Tobacco is a risk factor for many cancers including lung cancer, many diseases such as ischemic heart disease, cerebrovascular disease, chronic obstructive pulmonary disease, periodontal disease and pregnancy-related abnormalities. The components of tobacco smoke that affect the body include not only nicotine and tar but also many harmful substances such as carbon monoxide. In a case-control study in Sweden, smoking was a risk factor for CD but was not associated with snus use, so the association between smoking and CD is a virulence mechanism that is not nicotine from burning tobacco was stated [10]. Studies examining the effects of smoking on the disease course of CD did not show the effect of active smoking, but passive smoking was harmful. Because nicotine metabolite levels are much lower in passive smokers than active smokers, it suggests that non-nicotine factors in tobacco smoke-contaminated environments were associated with CD [11]. The results of our study may have had the same effect as these studies. From the above, both active and passive smoking should be avoided to prevent the development and progression of CD.

In the present study, the OR for CD was significantly decreased in ever or current drinkers. On the other hand, two cohort studies in Europe showed that there is no evidence that there is an association between alcohol intake and UC or CD onset [10, 13]. A study in Taiwan has shown that alcoholism is a risk of IBD [16]. In case-control studies that examined the relationship between UC and drinking alcohol, a study in Japan showed that usual consumption of alcohol reduced the risk compared with less frequent use [17], a study in China showed that light alcoholic drinking had protective effect against UC and this effect disappeared when smoking was associated with light alcohol drinking [18]. Like these, although the results of studies on the association between inflammatory bowel disease and alcohol intake are not consistent, there are differences in the disease between CD and ulcerative colitis, and the evaluation of alcohol intake varied. Two cohort studies in Europe have assessed alcohol intake using total alcohol intake (g / day) [10, 13], but unfortunately, as we examined "yes or no of alcohol history", we could not compare with those studies. Thus, Detailed examination is necessary from now on.

In the present study, although the sex- and age-adjusted OR of past appendicitis was significantly increased, the OR in the multivariate model was not significant. It has not yet been determined whether a history of appendicitis surgery is a risk factor for CD [3–5]. There is a tendency for CD to be misdiagnosed as appendicitis, which is one of the differential diagnosis, because a definite diagnosis of CD is often difficult. Therefore, care must be taken in the interpretation of the results. In the present study, a history of appendicitis occurring more than 1 year earlier was defined as “appendicitis history”, and the possibility of reverse causality was excluded as much as possible.

In past studies, "family history of inflammatory bowel disease" has been reported as a factor related to CD [19]. However, we could not confirm the association between IBD family history and CD in the subjects of the present study. The prevalence of family history of IBD was significantly lower in China compared with the United States (China: 4.1%, United States: 39.3%) [6]. In a study targeting Japanese, there are reports that the disease characteristics of CD differ depending on the presence or absence of IBD family history [19].

The strengths of this research are the methodological advantages in its design. First, since the cases were confirmed using strict diagnostic criteria, there is little chance of misclassification of CD. Secondly, weakness of recall on lifestyle before the onset of disease could be approached to the minimum with the use of incident cases (newly diagnosed CD patients). In addition, in order to evaluate the association with environmental factors before disease as precisely as possible, we collected pre-illness information as much as possible.

However, the following limitations may be affecting the study results. First, despite the gender- and age-matched case-control studies, we used an unconditional logistic regression model. Originally, it was considered desirable to use a conditional multiple logistic regression model considering matching. So we assessed 172 people (40 pairs with case:control = 1:2, 26 pairs with case:control = 1:1) that could be analyzed using the conditional logistic model. In the multivariate analysis, the OR increase of BMI <18.5 kg/m^2^ remained marginally significant, and the OR increase with past appendicitis showed marginal significance. Although the confidence interval was widened for other factors, it showed the similar result as the unconditional logistic model. Therefore, we consider the use of the unconditional logistic model in the present study to be acceptable.

Second, although the results of this study were obtained after adjusting for potential confounders, there are the issues of residual confounding, i.e. other unmeasured factors may have influenced the validity of our results.

Third, in this study, it was not possible to examine those who became active smokers after passive smoking and those who became passive smokers after quitting smoking. Because we collected passive smoking information only from non-active smokers, we could not simultaneously adjust active smoking and passive smoking in our model. In this study, we have shown an association between passive smoking and the development of CD in nonsmokers.

In conclusion, a case-control study was conducted to clarify the factors associated with the development of CD in Japanese persons. Passive smoking may be associated with the development of CD. Further research is necessary to confirm these findings.
